# Avian Influenza A(H7N9) Virus Screening in Patients with Fever and Flu-Like Symptoms in a Tertiary Hospital in an Area with Confirmed Cases

**DOI:** 10.1371/journal.pone.0082613

**Published:** 2013-12-18

**Authors:** Chao Wu, Rui Huang, Jianjun Chen, Qin Gu, Bin Zhu, Jun Wang, Kui Zhang, Quanjiao Chen, Chaochao Xiong, Yong Liu, Jiequan Li, Yi-Hua Zhou, Yitao Ding

**Affiliations:** 1 Department of Infectious Diseases, Nanjing Drum Tower Hospital, Nanjing University Medical School, Nanjing, Jiangsu, China; 2 Department of Infectious Diseases, Nanjing Drum Tower Hospital Clinical College of Traditional Chinese and Western Medicine, Nanjing University of Chinese Medicine, Nanjing, Jiangsu, China; 3 Center for Emerging Infectious Diseases, Wuhan Institute of Virology, Chinese Academy of Sciences, Wuhan, Hubei, China; 4 Department of Intensive Care Units, Nanjing Drum Tower Hospital, Nanjing University Medical School, Nanjing, Jiangsu, China; 5 Department of Radiology, Nanjing Drum Tower Hospital, Nanjing University Medical School, Nanjing, Jiangsu, China; 6 Department of Emergency Medicine, Nanjing Drum Tower Hospital, Nanjing University Medical School, Nanjing, Jiangsu, China; 7 Department of Laboratory Medicine, Nanjing Drum Tower Hospital, Nanjing University Medical School, Nanjing, Jiangsu, China; 8 Nanjing Municipal Center for Disease Control, Nanjing, Jiangsu, China; 9 Jiangsu Key Laboratory for Molecular Medicine, Nanjing Drum Tower Hospital, Nanjing University Medical School, Nanjing, Jiangsu, China; Centers for Disease Control and Prevention, United States of America

## Abstract

Novel avian influenza A(H7N9) virus was isolated in fatal patients in Yangtze River Delta of China in March 2013. We aimed to screen the virus in febrile patients in a tertiary hospital in an area with confirmed cases. Throat-swab specimens collected from consecutive patients with fever (≥38°C) and flu-like symptoms from April 15 to April 25, 2013 were subjected to detect novel avian influenza A(H7N9) virus with real-time PCR. The clinical outcomes in the patients and close contacts were followed up. Of total 200 patients screened, one (0.5%) was positive for avian influenza A(H7N9) virus and 199 others were negative. The infected patient experienced respiratory failure and had diffuse infiltrates in the right lower lobe in chest CT images. He received symptomatic and antibacterial treatments as well as oseltamivir. His condition was substantially improved within three days after admission; avian influenza A(H7N9) virus was not detected after 5 days' antiviral therapy. The hemagglutinin inhibition test showed that the serum titers against avian influenza A(H7N9) virus increased from <1∶20 at the early phase to 1∶80 at the convalescent phase. Follow-up of 23 close contacts showed that none of them developed fever and other symptoms within two weeks. Our findings suggest that although the infection rate of avian influenza A(H7N9) virus in patients with fever and flu-like symptoms is rare, the screening is valuable to rapidly define the infection, which will be critical to improve the clinical outcomes.

## Introduction

During March 2013, a novel influenza virus of avian origin, influenza A(H7N9), was isolated from three patients with fatal severe respiratory disease in Shanghai and Anhui, both located in the Yangtze River Delta Region, east part of China [1]. Subsequent reports show that the virus was detected in more patients from the same areas, including Nanjing, the capital of Jiangsu Province [2]–[4]. Up to August 11, 2013, a total of 135 patients, including 44 deaths, were confirmed to be infected with the virus; most of them came from the Yangtze River Delta including Nanjing city [5].

According to the diagnostic criteria, a suspected case of avian influenza A(H7N9) is defined when a patient has fever, respiratory symptoms such as sore throat or cough, toxemia symptoms such as headache, malaise, or myalgia, and supportive epidemiologic evidence such as contact with poultries and living at areas where confirmed case was reported [6]. However, screening for avian influenza A(H7N9) virus in febrile patients attending the hospital in areas with confirmed cases is rarely reported [7].

Nanjing Drum Tower Hospital is a 2,400-bed tertiary medical center with 35 departments, including an emergency department with the fever clinic and an independent intensive care unit (ICU) with 51 beds and 6 isolation rooms. During late March and early April 2013, five patients who attended to our hospital were confirmed to have caught avian influenza A(H7N9) [3]. This had us to assess the incidence of avian influenza A(H7N9) virus in patients with fever flu-like symptoms, who attended the fever clinic of our hospital, a major general hospital in areas with confirmed cases of avian influenza A(H7N9).

## Materials and Methods

### Ethics statement

Written informed consent was obtained from all patients involved in this study. The study was approved by the Ethics Committee of Nanjing Drum Tower Hospital (EC20130411-1).

### Samples and data collection

During the late March and the early April 2013, several patients with high fever and server pneumonia were admitted to our hospital. On April 2, two of them were confirmed to be infected with avian influenza A(H7N9) virus by the Chinese Center for Disease Control and Prevention in Beijing; one more patient was confirmed the infection by the Jiangsu Provincial Center for Disease Control and Prevention in Nanjing on April 4 [3], [4]. Once these three patients were diagnosed, we wanted to start screening avian influenza A(H7N9) virus in patients with fever and flu-like symptoms who attended to our hospital. However, we did not start the screening until April 15 because of the short of detection agents at that time. We stopped the screening after April 25 because rare cases had been confirmed to be infected with avian influenza A(H7N9) virus within one week, the presumed incubation period, before April 25. With the throat-swabs specimens collectors, we collected the throat swab samples from 200 consecutive febrile (≥38°C) patients with flu-like symptoms when they first went to our hospital from April 15 to April 25.

Each swab was vigorously shaken in 0.5 mL of diethylpyrocarbonate treated water and squeezed on the inside surface of tube to release as much trapped virus as possible before the swab was taken out. Each of the samples was added with 1.0 mL TRIzol (Invitrogen, Life Technologies, USA), vortexed for at least 15 s, and then stored at −70°C.

### RNA extraction and detection of influenza A(H7N9) virus

The total RNA was extracted from samples in TRIzol according to the manufacturer's instructions. To increase the recovery rate of RNA, we added 0.5 µL glycogen (20 mg/mL) in the precipitation of RNA. The final pellet was dissolved in 10 µL diethylpyrocarbonate treated water. Five microliters each was used to detect H7 and N9 genes respectively with the fluorescence reverse transcription (RT) PCR Detection kits (BioPerfectus Technologies, Taizhou, Jiangsu Province, China) on ABI 7500 Real time PCR system (Applied Biosystems). The protocols and primers were prepared in accordance with those provided the WHO Collaborating Centre in Beijing [1].

### Virus isolation

The virus isolation was performed essentially as described previously [8]. Briefly, throat-swab specimens taken from patients were maintained in viral transport medium on ice fewer than 24 hours, followed by filtration sterilization and inoculation into the allantoic cavities of 10-day-old specific-pathogen-free (SPF) embryonated eggs (Beijing MERIAL Ltd.). After incubation at 35°C for 48–72 h, the allantoic fluid of the inoculated eggs was collected and identified by hemagglutination assay.

### Hemagglutinin inhibition assay

The hemagglutinin inhibition (HAI) assay was performed with 0.5% chicken red blood cells as described elsewhere [9] with some modifications. Briefly, the sera from patients were treated with receptor-destroying enzyme (Denke-Seiken, Japan) and inactivated at 56°C for 30 min, followed by incubation with chicken erythrocytes to adsorb nonspecific agglutinins. Then the sera, starting at a 20-fold dilution, were 2-fold serially diluted with PBS, and added with 25 µL/well on a 96-well polystyrene microtiter plate, followed by adding 25 µL of virus suspension containing 4 hemagglutinin units per well. After incubation of the plate at room temperature for 1 hour, 50 µL of 0.5% (v/v) chicken red blood cells was added to each well. After incubation at room temperature for 30 minutes, the HAI titers were determined as the reciprocal of the highest serum dilution that completely inhibited hemagglutination.

### Statistical analysis

Statistical analysis was performed using SPSS software version 13.0 (SPSS, Chicago, USA). We summarized the continuous variables as means and standard deviation. For categorical variables, the percentages of patients in each category were calculated.

## Results

### Patient characteristics

From April 15 to April 25, 2013, totally 5,009 patients attended to the outpatient emergency department of our hospital. Of them, 337 with fever ≥38°C were required to attend the fever clinic of our hospital. A total of 200 febrile patients, who also presented flu-like symptoms, were recruited in this study to have their throat-swab specimens tested for the presence of avian influenza A(H7N9) virus with fluorescence RT-PCR. Their general characteristics and results of routine blood tests are shown in Table 1.

**Table 1:** General characteristics and routine blood test results of the 200 febrile patients.

Variable	Value* (n = 200)
Age, years	34.13±14.88
Gender	
Male	110 (55.0)
Female	90 (45.0)
Symptoms	
Fever	200 (100.0)
Temperature, °C	38.72±0.56
Sore throat	149 (74.5)
Cough	95 (47.5)
Myalgia	90 (45.0)
Malaise	10 (5.0)
Headache	89 (44.5)
Routine blood testing	
Leukocyte count, ×10^9^/L	10.18±4.14
<4×10^9^/L	5 (2.5)
>10×10^9^/L	93 (46.5)
Lymphocyte count, ×10^9^/L	1.43±1.01
Neutrophil, ×10^9^/L	7.93±3.91
Hemoglobin, g/L	140.10±17.73
Platelet count, ×10^9^/L	180.25±46.37

%) or mean ± standard deviation. Values are no. patients (

One (0.5%) of the 200 patients showed positive in the tests of both avian influenza virus H7 and N9 in RT-PCR and 199 others (99.5%) were negative. Additional throat-swab specimens from the patient with positive avian influenza A(H7N9) virus RNA, which had been collected the next day, were retested with the same kit in two other independent laboratories; both laboratories reported positive. Thus the patient was diagnosed with the infection of avian influenza A(H7N9) virus. All other patients were excluded from the infection of the virus, and recovered after 1–3 weeks treatment.

### Illustration of the patient infected with avian influenza A(H7N9) virus

The patient diagnosed with the infection of avian influenza A(H7N9) virus was a 36-year-old man who lived in Xinyi city, which is located in north part of Jiangsu Province, 310 km away from Nanjing. He was a living poultry retailer and had transported living poultry from Xinyi city to Hangzhou city, the capital of Zhejiang Province, where sporadic avian influenza A(H7N9) occurred from February to April, 2013 [2], [4]. He had a known exposure history to live chicken within a week before the onset. He developed fever (38.4°C), non-productive cough, malaise, and myalgia on April 18, and his condition was deteriorated with the highest temperature 40.5°C and dyspnea on the subsequent days. The patient was referred to our hospital on April 22, 2013.

**Figure 1:**
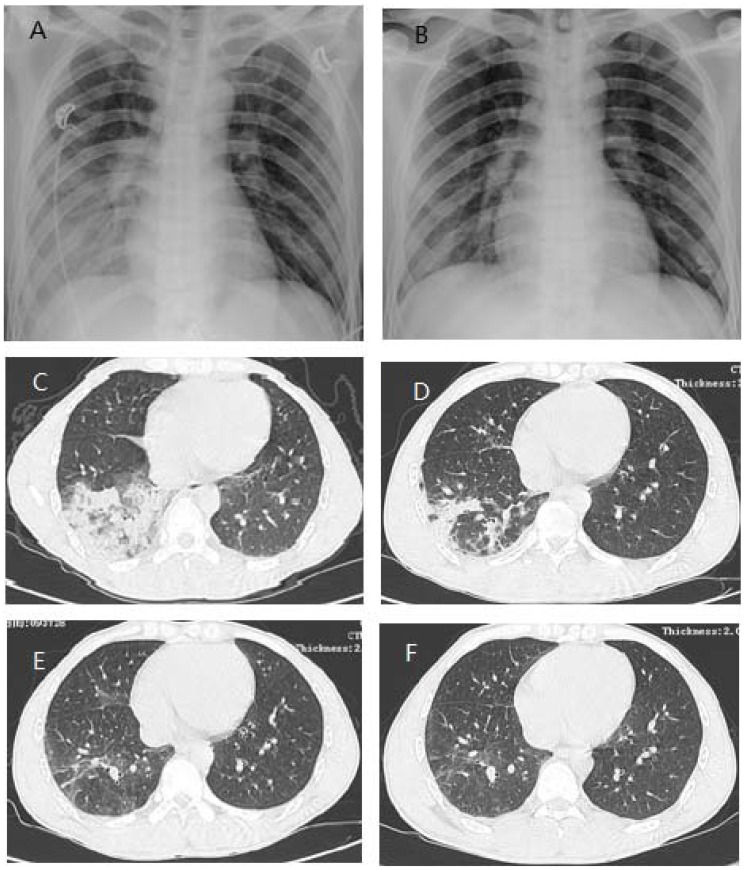
Chest radiographs and computed tomographic (CT) scans in the patient with avian influenza A(H7N9). (A and B) Chest radiographs taken 5 and 25 days respectively after the onset of symptoms. Diffuse infiltrates and consolidation may be observed in the right lower lobe on day 5 (A) and the infiltrates are largely disappeared on day 25 (B). (C to F) Chest CT scans during the illness course and follow-up. The scanning was performed on day 4 (C), 21 (D), 43 (E), and 75 (F) respectively after the onset of symptoms.

On the physical examination in our hospital, the patient had high fever (40.2°C), dyspnea and lip cyanosis. Blood tests revealed that leukocytes were 3.3×10^9^/L with neutrophils of 2.6×10^9^/L, lymphocytes of 0.5×10^9^/L, platelet of 93×10^9^/L, and red blood cells 4.38×10^12^/L, and the liver and kidney functions were generally normal. The sputum cultures for bacteria in the first three days were all negative. Arterial blood gas analysis presented the results of pH 7.441, PO_2_ 64 mmHg, PCO_2_ 30.5 mmHg, and SaO_2_ 93%, under nose tubular oxygen supplement (5 L/min). Chest x-ray and computed tomographic (CT) scanning revealed consolidation and diffuse infiltrates in the right lower lobe and patchy infiltrates in the left lower lobe (Figure 1A and 1C). He was diagnosed with severe pneumonia (suspected H7N9 influenza) and respiratory failure. He was admitted to the Respiratory Department, and given the oseltamivir (150 mg, twice daily) and antibiotics as well as other symptomatic treatment. Meanwhile, throat-swab samples were collected for detecting avian influenza A(H7N9) virus, which turned out to be positive on the test of April 23. On the next day, throat-swab samples were collected again for detecting avian influenza A(H7N9) virus; the viral RNA was positive in the two independent laboratories reported on April 24.

**Figure 2:**
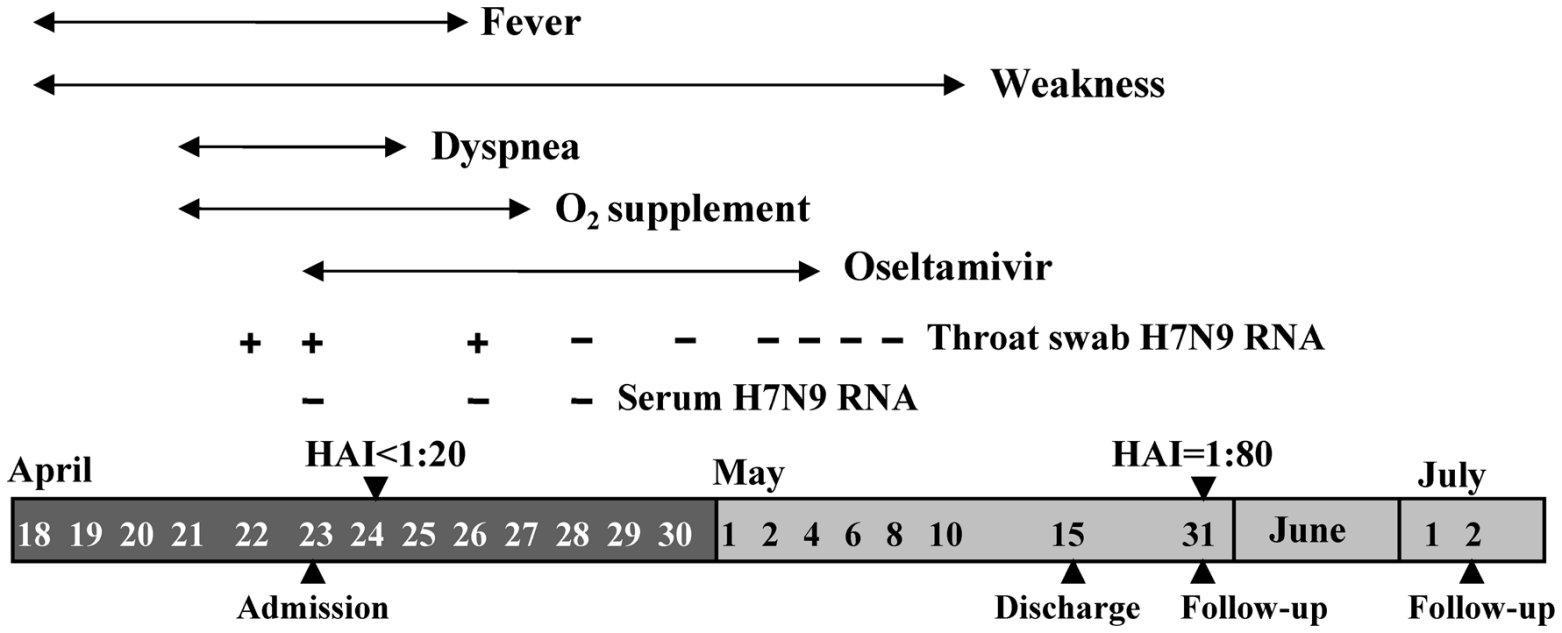
Course of the patient infected with novel avian influenza A(H7N9) virus. HAI, titers of hemagglutinin inhibition against the novel avian influenza A(H7N9) virus.

Once the diagnosis was established on April 24, the patient was quarantined in the isolation room to prevent the potential transmission and treated with the combination therapy against influenza A, including oseltamivir and piperacillin/tazobactam (Tazocin). The main clinical features, laboratory findings, and treatment during the disease course are presented in Figure 2. The patient received continuous nasal oxygen supplementation (2–5 L/min) in the period of April 23–April 25 and intermittent nasal oxygen supplementation (2 L/min) during April 26 and 27; he never used mask oxygen inhalation or mechanical ventilation. The patient's condition was substantially improved within three days after admission; the dyspnea disappeared and hypoxemia was corrected. The isolation of the virus from the throat-swab sample collected on April 25 was failed even after the sample was propagated in the allantoic sac and amniotic cavity of embryonated chicken eggs. Avian influenza A(H7N9) virus RNA in the throat-swab samples was still positive on April 26 but became undetectable on April 28 and remained negative thereafter, while the viral RNA was undetectable in the serum samples collected on the April 23, 26, and 28 respectively. The chest CT-scanning on May 9 showed that the patchy infiltrates in the left lower lobe disappeared and the diffuse infiltrates in the right lower lobe significantly improved (Figure 1D). In agreement with this, no consolidation was observed on the chest x-ray on May 13 (Figure 1B). On May 15, the patient was discharged after he had a good recovery. The follow-up on May 31 showed that the physical examinations were generally well and hematological and biochemical investigations did not reveal abnormal results, but his chest CT-scanning still had some abnormal in the right lower lobe (Figure 1E). A further follow-up conducted on July 2 revealed no abnormal findings in physical examinations, blood tests and chest CT-scanning (Figure 1F).

The hemagglutinin inhibition test showed that the serum at the early phase of the illness, on day 6 after the onset, had the titers lower than 1∶20 against avian influenza A H7N9, H5N1, and 2009H1N1, respectively, but had a titer of 1∶80 against seasonal influenza A H1N1. However, the serum of convalescent phase, on day 44 after the onset, had titers of 1∶80 against avian influenza A(H7N9) virus (Table 2).

**Table 2:** Hemagglutinin inhibition titers of the sera at different phases of the illness.

	Serum	
Virus isolate	Acute phase	Convalescent phase
A/Shanghai/2/2013 (H7N9)	<20	80
A/Anhui/1/2013 (H7N9)	<20	80
A/Chicken/Hunan/246/2012 (H5N1)	<20	<20
A/California/07/2009 (H1N1)	<20	20
A/New Caledonia/20/99 (H1N1)	80	80

### Contact tracing

The patient had not been quarantined until his diagnosis was established on April 24, 2013. Some persons closely contacted the patient without strict preventive measures. These included his parents, his wife and two children, and 18 physicians and nurses. We followed these persons to investigate whether avian influenza A(H7N9) virus was transmitted from human to human. None of the 23 close contacts developed the fever and flu-like symptoms within 14 days after contact.

## Discussion

In the present study, we investigated the novel avian influenza A(H7N9) virus in the patients with fever and flu-like symptoms in an area with confirmed cases during the onset season. Of the 200 patients investigated, one (0.5%) was defined with infection of the virus, indicating that, even in areas with the confirmed cases during the onset season, the infection rate of avian influenza A(H7N9) virus in the flu-like patients is low. However, the incidence is higher than that (0.03%) in 20,739 patients with influenza-like illness from 10 provinces with confirmed human cases [7]. The results that none of the 23 close contacts of the patient with influenza A(H7N9) developed flu-like symptoms suggest that the virus is not easily to transmit from human to human, which is in agreement with the other reports [10]–[12].

In this investigation, no patient with fever and flu-like symptoms who attended to the Emergency Department of our hospital during the screening period refused to participate, indicating that no patient infected with avian influenza A(H7N9) virus was neglected. Furthermore, the screening method, developed according to the protocols of the WHO Collaborating Centre in Beijing [1], is highly sensitive and effective in detecting the viral RNA in the throat-swab specimen [13]. Thus, it is less likely to underestimate the infection rate of avian influenza A(H7N9) virus in the patients with flu-like symptoms. Although the definitive etiological diagnosis were not clear, the benign outcomes of 199 other patients also suggested that they were not infected with avian influenza A(H7N9) virus. On the other hand, it is also impossible that the diagnosis of avian influenza A(H7N9) virus infection is a result of false positive, although the viral culture of throat-swab specimen from this patient was negative. The reasons supporting the diagnosis include: (1) the RNA of avian influenza A(H7N9) virus was detected in three independent laboratories; (2) the clinical features and disease course are in agreement with those of the patients with avian influenza A(H7N9); and (3) the most important, the hemagglutinin inhibition test against two isolates of the novel avian influenza A(H7N9) virus (Table 2) had the titers increased by >4-fold from the early phase to the convalescent phase.

Since avian influenza A(H7N9) virus causes fatal disease in human, one of main concerns on this disease is the issue of whether the virus is prone to human-to-human transmission. In the present study, we traced the close contacts who had not taken the precautionary measures, and none of them developed flu-like symptoms. The results imply that the human-to-human transmission of the avian influenza A(H7N9) virus appears to be rare, although it is reported that the virus can bind to human-type α2,6-linked sialic acid receptors, and infect epithelial cells derived from the human lower respiratory tract and type II pneumonocytes derived from the human alveoli [14]. Our data are consistent with the results of other studies [15], [16].

It is interesting to note that the patient infected with avian influenza A(H7N9) virus found in this screening had experienced the disease not as severe as most of the other reported patients [4]. In accordance with the benign course of the disease in this patient, the duration (8 days after the onset) of viral shedding in the throat swab specimens was also relatively short, compared with that reported elsewhere [13]. The relatively good clinical course in this patient may be attributed to several reasons. The first may be that the patient received the early treatment after the onset of the illness; this is attributed to the rapid response to the outbreaks of avian influenza A(H7N9) and the development and attribution of detection reagents. The second may be that the patient is much younger (36 years old) compared to those in a report with 111 patients (61 years old, median). Ip et al reported that 5 patients at a mean age of 13 years (range 2–26 years) had mild to moderate infection of avian influenza A(H7N9) virus, and three of them were even not hospitalized [16], suggesting that young persons may have benign prognosis than old ones. A more relevant example is that a 15-year-old boy appeared to have caught moderate avian influenza A(H7N9) and recovered after one week hospitalization; he was respectively diagnosed nearly after two months [17]. On the other hand, elderly people may have increased risk for H7N9 virus infection and have worse outcomes [10], [18].

In summary, although the infection rate of avian influenza A(H7N9) virus in an area with confirmed cases during the disease season is rare, the screening in patients with fever and flu-like symptoms is valuable to rapidly diagnose the patients in areas with confirmed cases during certain period of time, which will be critical to improve the disease outcomes and prevent the potential spread of avian influenza A(H7N9) virus.
